# Two step formation of metal aggregates by surface X-ray radiolysis under Langmuir monolayers: 2D followed by 3D growth

**DOI:** 10.3762/bjnano.6.247

**Published:** 2015-12-15

**Authors:** Smita Mukherjee, Marie-Claude Fauré, Michel Goldmann, Philippe Fontaine

**Affiliations:** 1 Sorbonne Universités, Université Pierre et Marie Curie (Paris 06), Institut des NanoSciences de Paris, CNRS-UMR 7588, 4 place Jussieu, 75005 Paris, France; 2 Faculté des Sciences Fondamentales et Biomédicales, Université Paris Descartes, 45 rue des Saints Peres, 75006 Paris, France; 3 Synchrotron SOLEIL, L’Orme des Merisiers, Saint-Aubin, BP 48, 91192 Gif-sur-Yvette, France

**Keywords:** GIXD, Langmuir monolayers, silver clusters, TXRF, X-ray radiolysis

## Abstract

In order to form a nanostructured metallic layer below a Langmuir monolayer, radiolysis synthesis was carried out in an adapted geometry that we call surface X-ray radiolysis. In this procedure, an X-ray beam produced by a synchrotron beamline intercepts the surface of an aqueous metal-ion solution covered by a Langmuir monolayer at an angle of incidence below the critical angle for total internal reflection. Underneath the organic layer, the X-ray beam induces the radiolytic synthesis of a nanostructured metal–organic layer whose ultrathin thickness is defined by the vertical X-ray penetration depth. We have shown that increasing the X-ray flux on the surface, which considerably enhances the kinetics of the silver layer formation, results in a second growth regime of silver nanocrystals. Here the formation of the oriented thin layer is followed by the appearance of a 3D powder of silver clusters.

## Introduction

Formation of metal nanoclusters and ultrathin metal–organic systems is an active research field. Indeed, due to their adjustable optical, magnetic, electronic, and catalytic properties these systems demonstrate many applications [1–2]. The usual approach for the synthesis of this type of material is the chemical reduction of metal-ion precursors. The radiolysis induced by reduction of metal ions is generally considered as an efficient method to control this synthesis in solution as it leads to the formation of monodisperse, tailored, metal nano-objects [3]. The synthesis route involves the irradiation of a metal-ion aqueous solution that induces the radiolysis of water. This results in the formation of various radicals (e.g., H^•^, HO^•^, e^−^_aq_). In the presence of a radical scavenger, H^•^ and e^−^_aq_ induce reduction of the metal ions into atoms that further aggregate in the solution to form metallic clusters. Usually γ rays or electrons [3] and more recently X-rays [4–5] are used to perform the irradiation.

In order to increase the variety of shapes of the formed nano-objects and to vary their properties, we have proposed a technique that brings together the advantages of both – the radiolytic reduction of metal ions to atoms and the self-assembly properties of surfactant molecules in solution. By the choice of the organic molecule, we can tune the surface charge of the formed self-assembly in order to attract the ions in their vicinity. Therefore, we expect that during the irradiation process, the aggregation of the reduced atoms takes place around the organic templates.

We have previously applied this strategy to a spherical and a planar geometry. In the first case, we observed the formation of silver nanoshells upon irradiation of an aqueous solution of linoleic acid micelles that contained silver ions [6–7]. In the latter case, we were able to form a dense, metallic silver layer anchored underneath an organic monolayer by irradiating with an X-ray beam in grazing incidence geometry, a Langmuir monolayer (mono-molecular layer of insoluble surfactant molecules) deposited on the free surface of a silver-ion aqueous solution. The thickness of the formed layer (here, 4.5 nm thick) created with this process is determined by the penetration depth of the X-ray evanescent wave [4]. Hence, we termed this method surface X-ray radiolysis. Based on the analysis of the intensity and the shape of the diffraction peaks that emerge in the spectra during the radiolysis process, we proved that the metal layer anchored to the organic monolayer consisted of silver atoms organized in thin crystallites oriented by the interface [4]. We also studied the kinetics of formation of this silver layer by total reflection X-ray fluorescence (TRXF) and grazing incidence off-specular scattering [8]. However, the X-ray source was the experimental limitation. The experiments were performed on the “difliq” beamline at Laboratoire pour l'Utilisation du Rayonnement Électromagnétique (LURE), which was a first generation synchrotron source with flux around 10^8^–10^9^ photons/s. Nowadays, third generation sources deliver about three orders of magnitude higher flux, at least 10^12^ photons/s.

In the present paper, we have used the new SIRIUS beamline equipped with a liquid surface diffractometer at the SOLEIL synchrotron [9] to perform X-ray surface radiolysis with a much higher X-ray flux and follow the evolution of the process for higher irradiation doses.

## Results and Discussion

**Figure 1:**
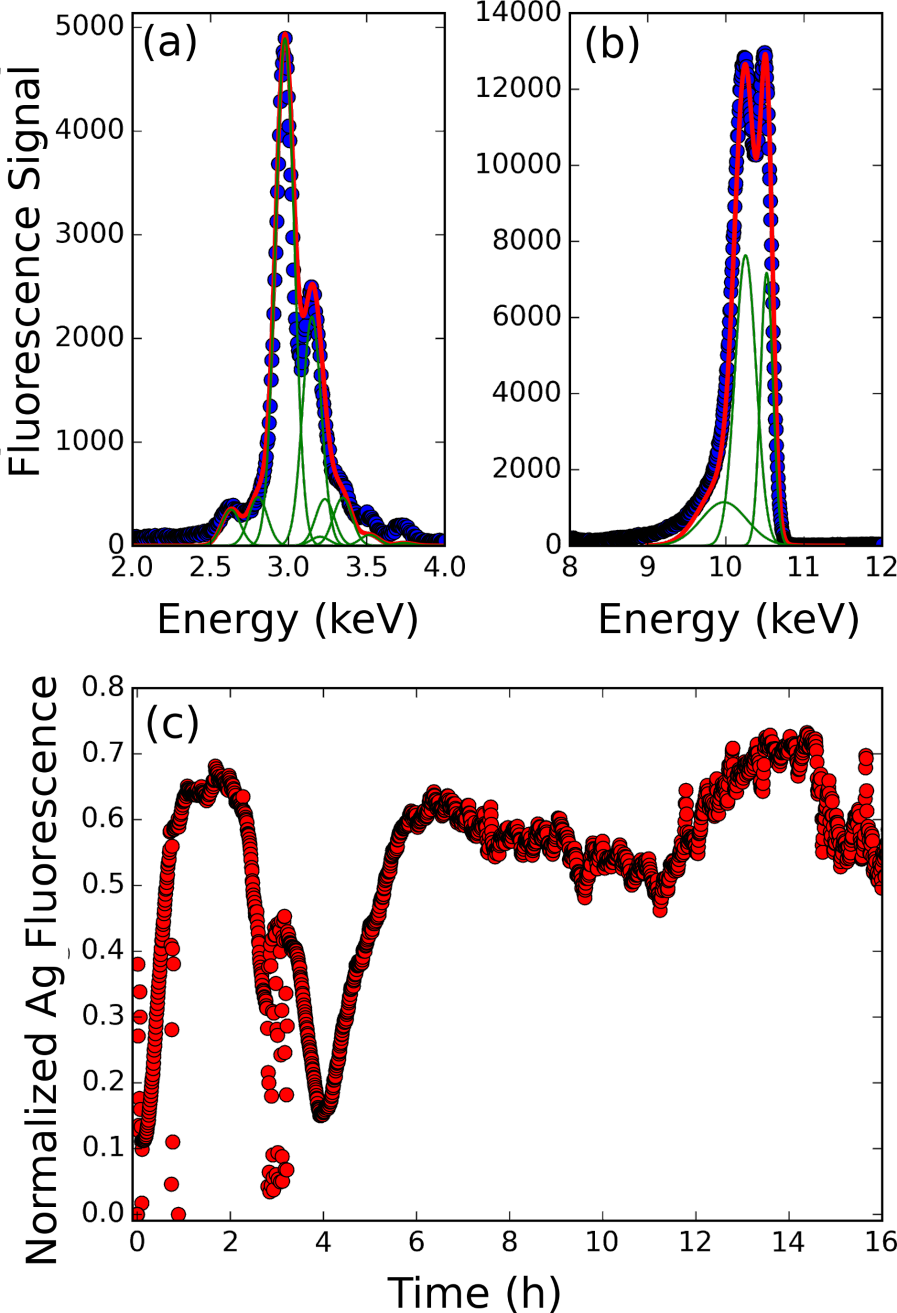
(a) X-ray fluorescence spectrum in the L-line energy range of Ag, where the green lines are the individual L line components, and the red is the fit by the sum of all lines. (b) Elastic and Compton peak analysis of the fluorescence spectrum. (c) Time evolution of the silver fluorescence intensity during 16 h of irradiation of a behenic acid Langmuir monolayer deposited on a silver sulphate aqueous solution. The signal is normalized by the elastic peak intensity.

Figure 1 presents the X-ray fluorescence measurement over the course of at least 16 h of irradiation for a behenic acid Langmuir monolayer deposited on silver sulphate and ethanol aqueous solution (alcohol is added as an HO^•^ radical scavenger), and compressed up to a surface pressure of 10 mN/m. The incident photon flux is approximately 10^12^ photons/s at the working energy 10.5 keV. In Figure 1a,b presents a typical fluorescence spectrum (split in two energy ranges) measured during the irradiation. Figure 1a is the L fluorescence emission due to the presence of silver atoms at the interface within the irradiated depth. The signal is analyzed by determining the weight of the individual fluorescence peaks, fitted by a Gaussian function, centered at each emission energy maximum, as reported in the literature [10]. Each time-stamped fluorescence spectrum is then fitted by the sum of these Gaussian functions weighted by the coefficients previously determined and a proportionality coefficient, *A*_f_(*t*). This coefficient is proportional to the amount of the excited element (here silver) in the irradiated volume. Therefore, its evolution reflects the change in the silver concentration. At high energy (Figure 1b), one observes the elastic peak and two peaks at slightly lower energy, which correspond to Compton scattering [11]. The three peaks are adjusted by a Gaussian function, where the elastic peak is centered at 10.5 keV and the intensity defines the *A*_e_(*t*) parameter for normalization.

The time evolution of the normalized fluorescence intensity, *A*_f_/*A*_e_ is depicted in Figure 1c. The first hours are characterized by an initial and fast increase of the fluorescence intensity for about 30 min after the irradiation started, followed by a constant fluorescence signal. The fluorescence intensity remains constant for about 2.5 h and then decreases suddenly down to almost its initial value at the beginning of the irradiation. Then, it rises up again up almost to its previous value but also shows strong fluctuations.

The first regime associated with the fluorescence intensity increase can be associated with an increase of the amount of silver atoms in the irradiated volume, defined by the X-ray footprint (1 × 50 mm^2^) and the penetration depth (4.6 nm) of X-rays. Comparing this curve to the result obtained from the previous experiment performed on a first generation synchrotron source with a much lower flux (10^9^ photons/s) [8], a distinct difference is first observed in the time needed to reach saturation. This is reduced from 5 h at the LURE down to 30 min at SIRIUS, demonstrating a significant increase of radiolysis kinetics, which is of course related to the increase of the dose rate directly related to the source intensity.

Such a reduction of the saturation time (one order of magnitude) makes the observation of the film transformation accessible when irradiation continues after the saturation is reached. The evolution exhibits two main features: a huge fluctuation ending with an increase back to the first saturated value of the fluorescence intensity, which is followed by a slow decrease and then increase resulting in a 15% fluctuation of the signal around the saturation value. This indicates the lack of a strong variation in the silver concentration in the probed volume in this second regime.

Two explanations can be proposed for the huge fluctuation: the first one is associated with the observation of a decrease of the surface pressure down to zero as the irradiation proceeds. One can then consider that the fluidity of the monolayer is enhanced, increasing the in-plane mobility of the patches of irradiated material. Thus, the surface region initially irradiated could drift in and out of the footprint area, leading to various thickness of the silver film and thus inducing the observed fluctuations. The second explanation is associated with the loss of silver patches (that can submerge into the sub-phase) followed by the formation of new ones by the incident beam.

**Figure 2:**
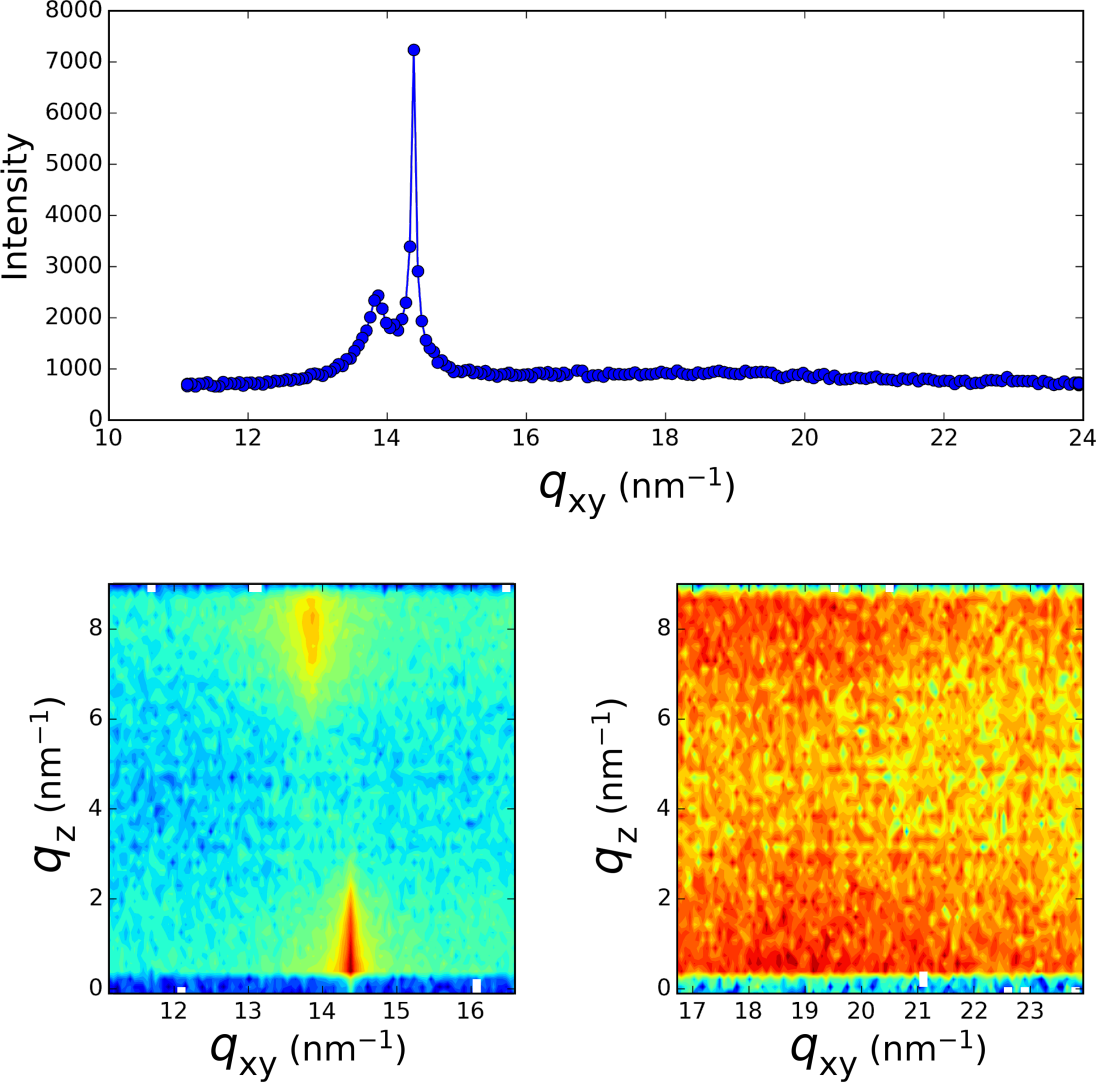
Grazing incidence X-ray diffraction spectrum taken at the beginning of the irradiation process of Figure 1c. Top: *q*_z_ integrated spectrum, Bottom: *q*_xy_−*q*_z_ intensity maps.

**Figure 3:**
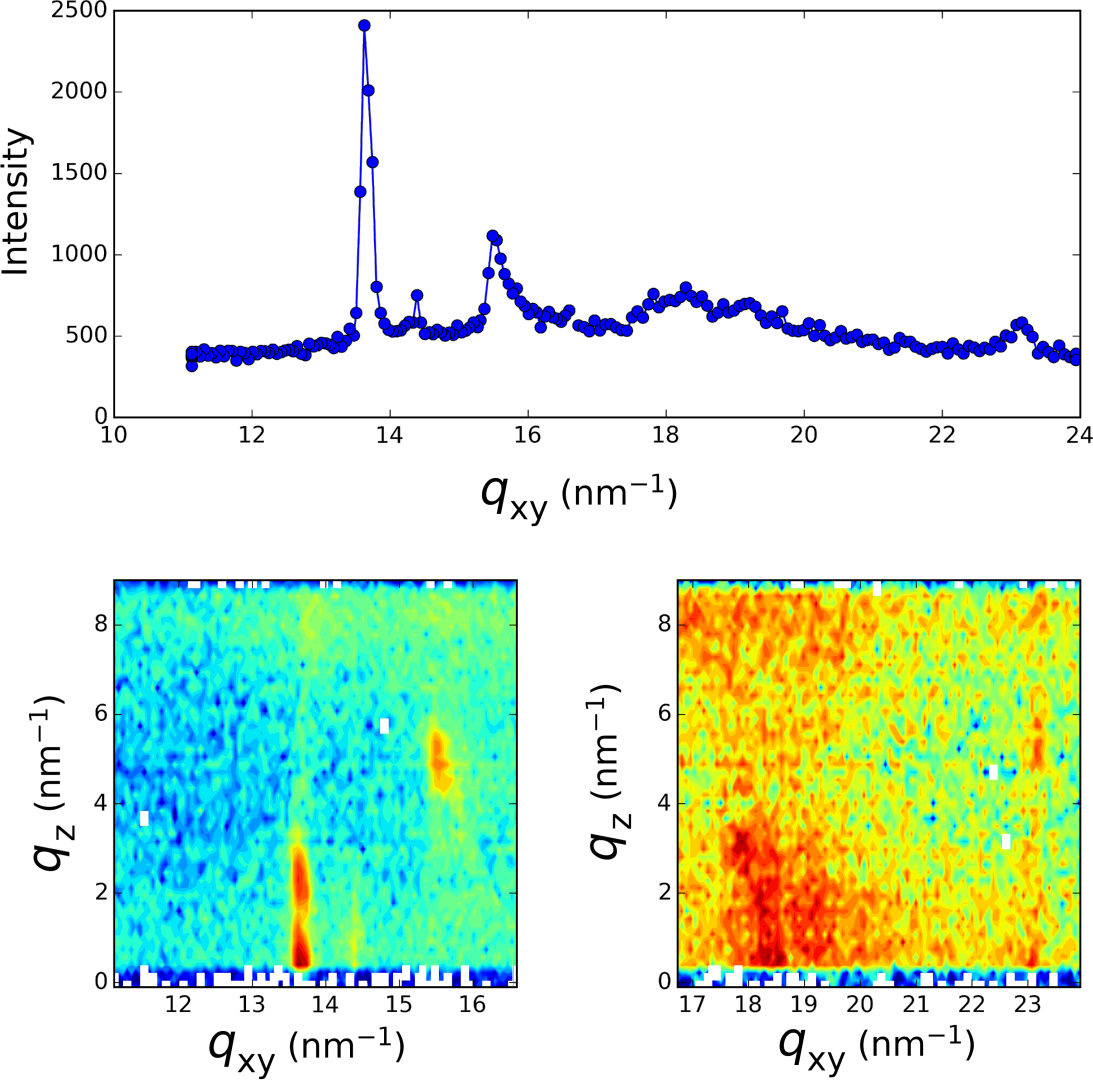
Grazing incidence X-ray diffraction spectrum taken after two hours into the irradiation process of Figure 1c. Top: *q*_z_ integrated spectrum, Bottom: *q*_xy_−*q*_z_ intensity maps.

**Figure 4:**
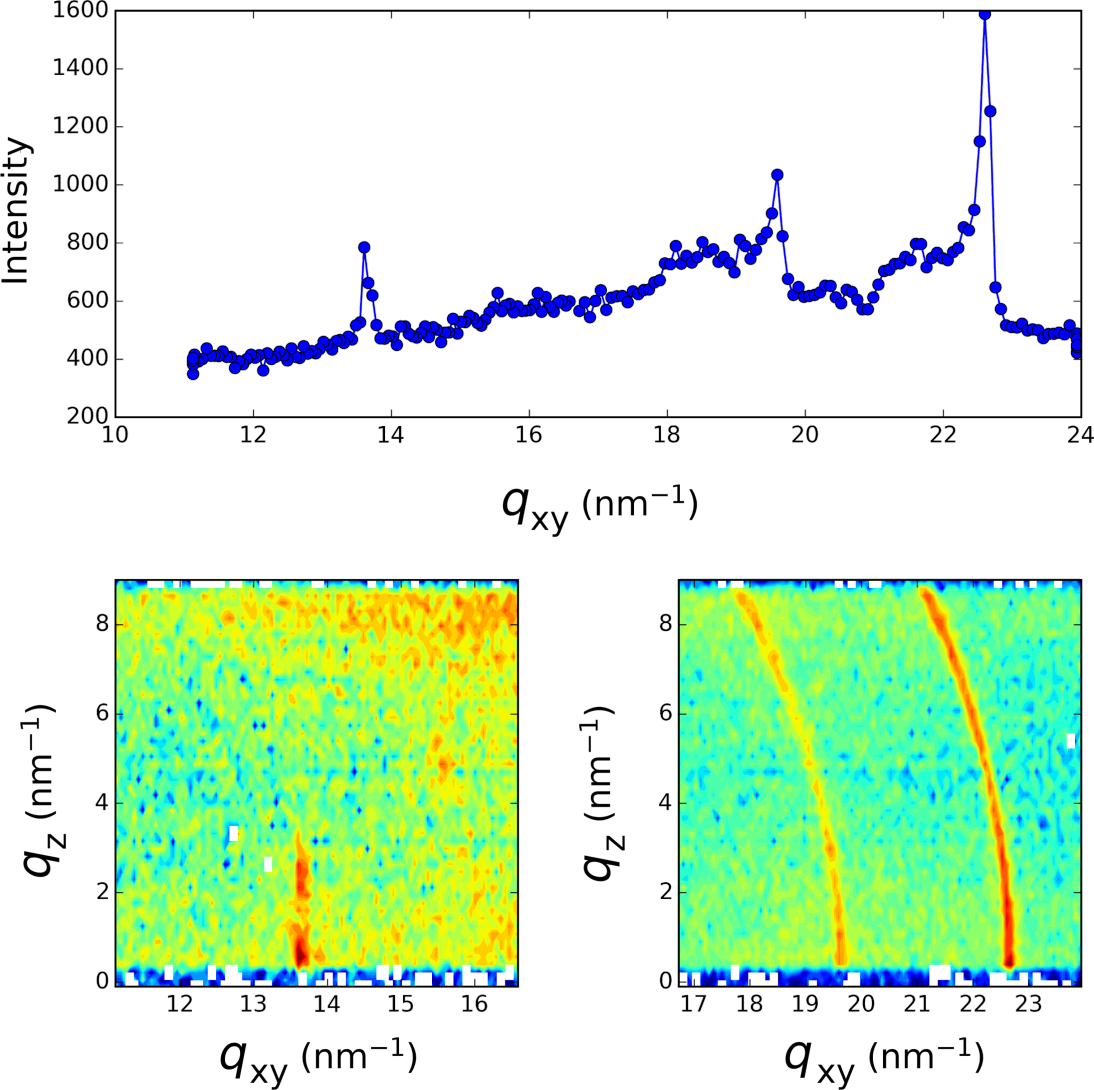
Grazing incidence X-ray diffraction spectrum taken after 15 h into the irradiation process of Figure 1c. Top: *q*_z_ integrated spectrum, Bottom: *q*_xy_−*q*_z_ intensity maps.

In order to obtain information about the structure of the formed layer at the air–water interface, we record the fluorescence signal (TRXF) simultaneously with the grazing incidence X-ray diffraction signal (GIXD) over a broad *q*-range covering the scattering wave vector transfer for the expected diffraction peak of 2D and 3D silver crystals. Figure 2, Figure 3 and Figure 4 present the diffraction spectra at different times over the course of the 16 h irradiation period of Figure 1. During the first 30 min of irradiation, the evolution previously measured at the LURE was recovered [4,8]. The two peaks (one in-plane at 14.38 nm^−1^ and one out-of-plane at 13.87 nm^−1^) assigned to the L2 phase of Langmuir monolayers of fatty acids [12] are observed at the beginning of the irradiation (Figure 2). Upon further irradiation, these peaks vanish (as the surface pressure decreases) and two new peaks located at *q*_xy_ = 13.65 nm^−1^ and *q*_xy_ = 15.52 nm^−1^ appear whose intensity grows with time (Figure 3). The *q*_z_ profile of these peaks appears as is characteristic of a 2D powder, that is, the crystallites present the same lattice plane parallel to the surface but random in plane orientation. This result is identical to that previously observed [4]. However, in the *q-*range of the 3D silver structure, the appearance of weaker peaks is observed, although they are presented as vertical rods characteristic of a 2D structure.

At longer time (*t* > 30 min), the lower *q*-range only exhibits some intensity fluctuations of the 2D diffraction peaks. The more striking feature is the appearance and growth in the high *q*-range of two diffraction rings for *q* = 19.60 nm^−1^ and *q* = 22.64 nm^−1^ (Figure 4). Such rings are characteristic of a 3D powder. These *q-*values do not correspond to that expected for the structure of bulk silver crystals, which are located at higher *q-*values (e.g., 26.6 nm^−1^ for the 111 reflection). However, smaller *q*-values corresponding to a larger feature size have been reported for small silver clusters exhibiting a crystal structure different from fcc [13–14]. The final picture of the system (after 16 h of irradiation) results in the co-existence of a 2D structure that grows rapidly after the initial irradiation and a 3D crystal powder that appears after the completion of the formation of the 2D layer at a longer irradiation time.

## Conclusion

Increasing the photon flux allows us to probe the formation kinetics of the inorganic film at longer time scales when irradiating the surface of a silver solution covered by a Langmuir monolayer. We evidenced that the growth of silver crystal continues with transformation of the film structure, even after the saturation concentration of the silver atoms is reached in the irradiation volume. However, in this second step, one observes the growth of clusters that are no longer oriented by the interface, as demonstrated by the appearance of a 3D powder. The key point of this study is that the appearance of late 3D silver crystal growth seems only to appear in the fluorescence signal with an increase of the fluctuations. This indicates that, under irradiation, these disoriented crystallites replace the previously oriented ones. We underline that the probed thickness is defined by the penetration depth of the X-ray evanescent wave, and thus, the silver film can be thicker for the latter case. This could be related to the observation of the evolving FWHM of the 2D peaks under irradiation. We previously observed [8] that the in-plane coherence length of the 2D crystal growth (deduced form the FWHM) is continuous under irradiation, even after the saturation of the fluorescence intensity is reached. This indicates that when the irradiated volume is completely filled with oriented 2D crystals, the irradiation induced the in-plane coalescence of these 2D crystals. One then obtains a 2D crystal with a surface area of approximately 10 µm^2^ with thickness of about 4.5 nm (equal to the X-ray penetration depth) [4]. However, the surface energy of the two surfaces of this platelet should differ since the interfaces are different (water/Ag below and organic molecule/air above). Such difference should induce a curvature of the platelet, which could rupture when the surface energy reaches a critical value. This could lead to the disorientation of the platelets and the formation of the 3D structures, which are then thicker than the X-ray penetration depth.

## Experimental

The sample preparation procedure was previously described in detail [4,8]. Droplets of 5 mmol·L^−1^ of behenic acid solution (C_21_H_43_COOH, Sigma-Aldrich) in chloroform (CHCl_3_) were spread onto the silver-ion solution in a Langmuir trough of 700 cm^2^ area and 1 cm depth. The temperature of the subphase was maintained at *T* = 20 ± 1 °C. The monolayer was slightly compressed up to π = 10 mN·m^−1^, the surface pressure at which the irradiation process is started. The Langmuir trough is enclosed in a gas-tight box flushed by helium gas. This allows the deoxygenation of the aqueous phase over the course of the experimental time scale and prevents bulk oxygenation by air during the radiolytic process.

The monolayer subphase is made by dissolving silver sulfate, Ag_2_SO_4_ (Sigma-Aldrich, purity 99%), in ultrapure water (Millipore system, 18 MΩ·cm) at a silver concentration of 1.5·10^−4^ mol·L^−1^. Ethanol (CH_3_CH_2_OH) was added at a concentration of 0.2 mol·L^−1^ as a radical scavenger [15]. Indeed, irradiation of deoxygenated water leads to the formation of three radicals, HO^•^, H^•^ and hydrated electrons, e^−^_aq_. By addition of ethanol to the aqueous solution, HO^•^ radical species are readily scavenged and lead to secondary radical creation, CH^•^_3_CH_2_OH. The remaining e^−^_aq_, H^•^ and CH^•^_3_CH_2_OH are strong reductive agents and thus induce silver-ion reduction into atoms, which coalesce and finally form the silver aggregates.

The X-ray irradiation and scattering measurements were performed on the SIRIUS beamline at the SOLEIL synchrotron. The details and optics of the facility are described elsewhere [9]. The incident X-ray energy used was 10.5 keV (λ = 0.118 nm) and the beam size was 0.1 × 1 mm^2^ (V × H) at the sample position. The water surface was illuminated at an incident angle of 1.8 mrad below the critical angle of the air–water interface (2.04 mrad at 10.5 keV), so that the incident wave was totally reflected, while the refracted wave became evanescent, exploring a layer of several nanometers beneath the interface. The scattered intensity was collected on a very low noise, position sensitive, 1D gas detector, with 2048 channels on 150 mm. A custom-built Langmuir trough was enclosed in a temperature-controlled, sealed chamber and flushed with helium during data collection to reduce gas scattering and to avoid beam damage to the monolayer. GIXD was used to obtain in-plane information about the molecular structure of the surface. The spectra were obtained by varying the X-ray, momentum transfer, in-plane component *q*_xy_ that is parallel to the air–water interface. The scattered intensity was measured as a function of the angle, 2θ, between the incident and diffracted beam projected onto the horizontal plane.

The X-ray fluorescence signal was measured using a one-element, silicon drift detector (Brüker, Germany) equipped with a collimator and mounted at 30° with respect to the vertical direction towards the X-ray source in order to reduce the elastic peak that would otherwise saturate the detector.
